# A New Bat-HKU2–like Coronavirus in Swine, China, 2017

**DOI:** 10.3201/eid2309.170915

**Published:** 2017-09

**Authors:** Lang Gong, Jie Li, Qingfeng Zhou, Zhichao Xu, Li Chen, Yun Zhang, Chunyi Xue, Zhifen Wen, Yongchang Cao

**Affiliations:** State Key Laboratory of Biocontrol, Life Sciences School, Sun Yat-sen University, Guangzhou, China (L. Gong, J. Li, Z. Xu, L. Chen, Y. Zhang, C. Xue, Z. Wen, Y. Cao);; Guangdong Wen’s Foodstuffs Group Co., Ltd., Yunfu, China (J. Li, Q. Zhou, L. Chen, Z. Wen)

**Keywords:** swine, diarrhea, coronavirus, Coronaviridae, bat-HKU2, porcine enteric alphacoronavirus, PEAV, viruses, zoonoses, China

## Abstract

We identified from suckling piglets with diarrhea in China a new bat-HKU2–like porcine coronavirus (porcine enteric alphacoronavirus). The GDS04 strain of this coronavirus shares high aa identities (>90%) with the reported bat-HKU2 strains in *Coronaviridae*-wide conserved domains, suggesting that the GDS04 strain belongs to the same species as HKU2.

Several pathogens are thought to be responsible for porcine diarrhea, including porcine epidemic diarrhea virus (PEDV) (*1*), transmissible gastroenteritis virus (*2*), porcine deltacoronavirus (*3*), porcine group A rotavirus (*4*), and emerging viruses like porcine kobuvirus (*5*). To add to the list, we have identified from suckling piglets with diarrhea in China a new bat-HKU2–like porcine coronavirus (porcine enteric alphacoronavirus [PEAV]).

Since December 2010, large-scale outbreaks of diarrhea in suckling piglets have been reported across China (*1*), and vaccination against PEDV has been relatively effective for diarrhea prevention. However, in February 2017, outbreaks of severe diarrhea occurred in swine herds vaccinated against PEDV in Guangdong, China. All ill pigs showed severe watery diarrhea, and their clinical onset occurred a few days later than those infected with PEDV. In initial tests with reverse transcription PCR using specific primers for PEDV, transmissible gastroenteritis virus, porcine group A rotavirus, or porcine deltacoronavirus, none of these viruses could be detected in all clinical samples. Furthermore, the recovered sows showed no seroneutralizing antibodies against PEDV.

To investigate the possible causative pathogen or pathogens causing this recent severe diarrhea in suckling piglets, we obtained excreta from 32 ill newborn piglets from 3 farms. We divided 5-day-old piglets into 4 groups of 5 each (3 groups according to the origin of the excreta, plus 1 control group). We inoculated each animal with 5 mL of excreta through the oral route. After 2 days, all inoculated animals exhibited similar clinical symptoms, including diarrhea and dehydration. We randomly selected 2 inoculated pigs in each group and performed necropsies on days 3 and 5 postinoculation, respectively. We filtrated homogenate of small intestine and intestinal contents, using the resultant supernatant for RNA extraction as described previously (*2*). We extracted total RNA by using a TRIzol reagent (Invitrogen Life Technologies, Grand Island, NY, USA) and eliminated ribosomal RNA with the Ribo-Zero rRNA Removal Kit (Illumina, San Diego, CA, USA) according to the manufacturer’s instructions. After reverse transcription PCR with random primers, we performed 150-bp paired-end shotgun metatranscriptome sequencing on the cDNA libraries by using an Illumina HiSeq system.

After assembling and mapping sequencing reads, we obtained a complete genome sequence of the PEAV GDS04 strain, which we then deposited in GenBank (accession no. MF167434). The full length of the PEAV genome is 27,171 nt (excluding the poly-A tail); this size is similar to that of bat-like HKU2 strains of coronavirus (*6*). The full genome of PEAV GDS04 strain shares high nucleotide identities (≈95%) with the reported bat-HKU2 strains.

Our phylogenetic analysis based on the whole genome of GDS04 and representatives of 4 established coronavirus genera, including the human and bat-like coronaviruses, demonstrated that GDS04 clusters with bat-like coronaviruses (Figure). According to the phylogenetic tree, the position of GDS04 is between HKU2 and BtRf alphacoronavirus. The HKU2 strain was identified in Hong Kong and Guangdong Province, China, in 2007 (*6*), and the BtRf alphacoronavirus strain was detected in China in 2012 (http://www.ncbi.nlm.nih.gov/nuccore/NC_028824.1). Both viruses are bat-associated viruses. However, the 2 strains are distributed in relatively different branches (Figure). Based on a comprehensive comparative analysis of the genomes of various groups of coronaviruses, we classified GDS04 as an alphacoronavirus. Our sequence analysis revealed that GDS04 has 80% nt and 87% aa identity with the spike (S) protein of the HKU2 strain. Moreover, the S protein of the GDS04 genome is 6 bp longer than the S protein in the HKU2 strain, which has the smallest S protein among all coronaviruses. Nevertheless, the GDS04 strain shares 95.7% aa identity with HKU2 in nonstructural protein (nsp) 3 (adenosine diphosphate-ribose 1′-phosphatase), 96.4% in nsp5 (3C-like protease), 94.6% in nsp12 (RNA-dependent RNA polymerase), 99.8% in nsp13 (helicase), 99.2% in nsp14 (3′-to-5′ exonuclease), 99.4% in nsp15 (poly[U]-specific endoribonuclease), and 97.6% in nsp16 (2'-*O*-ribose methyltransferase). These nonstructural proteins are *Coronaviridae-*wide conserved domains in replicase polyprotein pp1ab. A threshold of >90% in aa sequence identity suggests that 2 viruses are of the same species; our findings suggest that the GDS04 strain belongs to the same species as HKU2.

**Figure:**
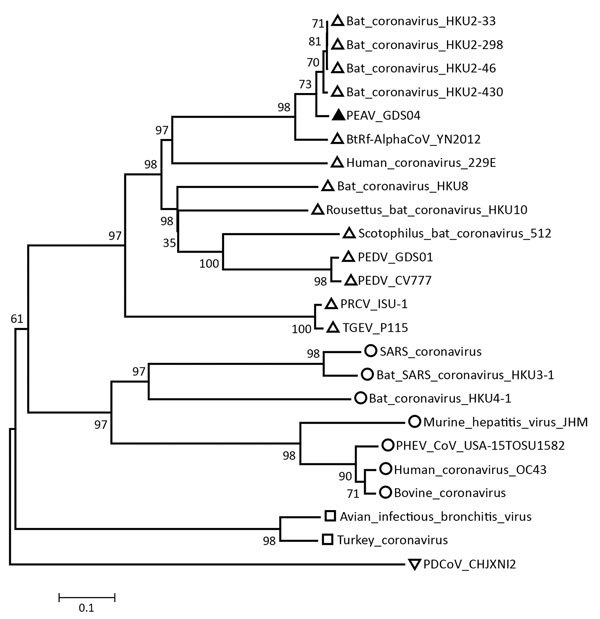
Phylogenetic tree based on the whole-genome sequences of PEAV, bat CoVs, and other representative CoVs, China, 2017. Analyses were conducted by using MEGA software version 6.0 (http://www.megasoftware.net) with the neighbor-joining algorithm. Bootstrap values were calculated with 1,000 replicates. The number on each branch indicates bootstrap values. Solid triangle indicates the GDS04 strain, open triangles alphacoronaviruses, circles betacoronavirusese, squares gammacoronaviruss, inverted triangle deltacoronavirus. Scale bar indicates nucleotide substitutions per site. CoV, coronavirus; PDCoV, porcine deltacoronavirus; PEAV, porcine enteric alphacoronavirus; PEDV, porcine epidemic diarrhea virus; PHEV, porcine hemagglutinating encephalomyelitis virus; PRCV, porcine respiratory coronavirus; SARS, severe acute respiratory syndrome; TGEV, transmissible gastroenteritis virus.

Furthermore, we designed specific primers of the n gene for the detection of the GDS04 strain. By using reverse-transcription PCR with these primers, we found that 97 out of 308 clinical intestinal or fecal samples were positive for GDS04. We collected all clinical samples from 25 farms in Guangdong Province during February–April 2017 and used 20 samples collected from healthy vaccinated piglets as negative controls.

In summary, we report preliminary data on our detection of a new coronavirus-like virus, PEAV. PEAV is thought to be responsible for the most recent diarrhea endemic in pig herds in southern China. Virus isolation and serologic testing are underway. The outbreak of the newly discovered virus arose among swine with severe diarrhea in swine breeding farms in southern China, suggesting the regional outbreaks of diarrhea could contribute to the emergence of new pandemic viruses. Extensive surveillance for GDS04 PEAV is required to define its epidemiology and evolution.
